# Identification and Characterization of New Seedborne Pathogens in *Phaseolus vulgaris* Landraces of Southern Italy

**DOI:** 10.3390/pathogens12010108

**Published:** 2023-01-09

**Authors:** Eliana Dell’Olmo, Massimo Zaccardelli, Boris Basile, Giandomenico Corrado, Loredana Sigillo

**Affiliations:** 1 Council for Agricultural Research and Economics (CREA), Research Centre for Vegetable and Ornamental Crops, 84098 Pontecagnano, Italy; 2 Department of Agricultural Sciences, University of Naples Federico II, 80055 Portici, Italy

**Keywords:** *Phaseolus vulgaris*, seed, germplasm, landrace, *Botryosphaeriaceae*, seedborne pathogens

## Abstract

The diagnostic survey of seedborne fungal pathogens is fundamental for symptomless material stored in gene banks to avoid the diffusion of pathogens by germplasm distribution and propagation. In this work, seeds of Southern Italian landraces of the common bean (*Phaseolus vulgaris* L.) belonging to the gene bank at CREA (Italy) were inspected to assess their phytosanitary status. The phytopathological analysis revealed the presence of the most common pathogens associated with common bean seeds such as *Fusarium* spp., *Macrophomina phaseolina*, *Rhizoctonia solani*, *Colletotrichum lindemuthianum* and *Diaporthe*/*Phomopsis* complex. However, new fungi able to completely inhibit seed germination were also observed. The most aggressive were isolated, and the morpho-pathological characterization, DNA sequencing and phylogenetic analysis allowed us to define the strains as *Botryosphaeria dothidea* CREA OF 360.4 and *Diplodia mutila* CREA OF 420.36. These two plant pathogens are generally associated with grapevines and other fruit trees. Pathogenicity tests were carried out along with a transmissibility test in which the transmission of the pathogens to the seedlings was proven. Host range experiments revealed the ability of these pathogens to infect crops such as pepper and melon. To our knowledge, this is the first time that *B. dothidea* and *D. mutila* were detected on the common bean.

## 1. Introduction

Legumes play an important role in the human diet since they have beneficial effects on health and are rich in vitamins, minerals and proteins [1,2]. Among them, the common bean (*Phaseolus vulgaris* L.) is one of the most cultivated food crops in the world [3]. Given the adaptability of this legume to environmental conditions, the common bean is cultivated in all geographic regions, including South America, Europe and Africa. In Italy, common bean cultivation occupies about 24,000 ha [3] and in the Campania region (Southern Italy) there is a high number of landraces with different morphological and nutritional features [4]. In the past years, the common bean landraces have been largely neglected because usually they are grown only in small areas to be sold in local markets [5]. This germplasm is increasingly valued since it can be used as a source of biodiversity and genetic variability [6]. To protect this material, in situ and ex situ collections were implemented in the Campania region as well as in other Italian regions such as Basilicata, Apulia and Veneto [3,6,7]. Common bean landraces have been characterized from an agronomic point of view, to understand the most suitable cultivation conditions [8]; morphologically, because this variability can be exploited to improve several traits such as seed color and shapes; nutritionally, for vitamins, proteins and other high quality nutrients [9]; and genetically, in order to verify the genotype and the genetic differences among the varieties [10].

In the framework of the Rural Development Program promoted by the European Commission to safeguard agricultural biodiversity, many efforts have been carried out to preserve landraces. Actions range from their collection to the possible exploitation through pre-breeding. In the recovery phase, importance should be given to the screening of pathogens in the propagation material. Seedborne pathogens are a serious threat to agriculture and germplasm collections, as they can affect seeds directly or indirectly [11]. In the first case, fungus and bacteria can colonize the seeds inducing visible symptoms and causing the loss of the grain value on the market. On the other hand, seedborne pathogens can remain in the seeds, and inhibit their germination. Subsequently, they can be transmitted to the seedling causing its death or damaging the roots and the vascular system, with yield reduction and strong economic loss [12]. Moreover, seeds are considered the first causal agent in the dissemination of novel pathogens in areas where they were not present before, representing a risk for other crops [13,14,15].

The most common seedborne fungi associated with *P. vulgaris* are *Alternaria* spp., *Penicillium* spp. and *Cladosporium* spp., which are considered external contaminants, while *Colletotrichum lindemuthianum* (Sacc. and Magn.) Briosi et Cav. [16], *Macrophomina phaseolina* (Tassi) Goid [17], *Rhizoctonia solani* (J.G. Kühn) [18], *Fusarium* spp. And *Diaporthe*/*Phomopsis* complex are the key pathogens of this species [3,19]. Climate changes and global warming have led to the outbreak of new diseases caused by fungal pathogens never detected before [3]. For example, the negative impact of the complex fungal family of *Botryosphaeriaceae* in agriculture can be aggravated in relation to more stressful climatic conditions. This family is composed of pathogens that usually infect woody plants, but they are characterized by polyphagous behavior that allow them to have a wide host range including vegetable crops especially under stressful conditions [20]. In addition, the ability of *Botryosphaeriaceae* to behave as seedborne pathogens has been also previously described [21]. For instance, *Lasiodiplodia theobromae* (Pat.) Griffon and Maubl. [22,23] is the causal agent of the black seed rot that devastated the pines in North America and was reported as a pathogen of beans in Nicaragua [15,21].

In this work, a phytopathological screening was carried out on common bean seeds to assess the presence of well-known saprophytes and pathogens, but also to investigate the presence of novel species and their effects on germination and seedlings development. Specifically, we carried out the phytopathological screening of seeds of 38 accessions of *P. vulgaris*. The morphological characterization of isolates allowed us to discriminate the commonly spread contaminants but also revealed emerging pathogens belonging to the *Botryosphaeriaceae* family. The novel contaminants were morphologically characterized and identified by molecular analyses. A pathogenicity test was performed to confirm the compatibility of *Botryosphaeriaceae* strains with *P. vulgaris*. Moreover, the host range analysis was carried out to confirm the pathogenicity on hosts already reported in the literature, and to explore the behavior of the isolated pathogens on related species and vegetables of economic relevance in Italy. Finally, phylogenetic analyses were also conducted. To the best of our knowledge, this is the first description of *Botryosphaeriaceae* affecting *P. vulgaris* seeds.

## 2. Materials and Methods

### 2.1. Seeds Collection and Mycological Analyses

Seeds of 38 local varieties of common bean (*Phaseolus vulgaris* L.) in the gene bank at the Council for Agricultural Research and Economics—Research centre for Vegetable and Ornamental Crops (CREA OF, Pontecagnano, Italy) were collected and stored within the activity of the “AgroBiodiversità Campana” project, funded by the “Regione Campania”, under the Rural Development Program 2014–2020 Measure 10.2.1. Seed analysis was carried out using the “7016: Detection of *Phomopsis* complex in *Glycine max* (L.) Merr. (soybean, soya bean) seeds” protocol as described in the International Rules for Seed Testing (ISTA) methods [24] with some modification. Briefly, 100 seeds for each sample were surface sterilized by soaking in sodium hypochlorite (1% *v*/*v*) for 10 minutes with gentle rotary shaking, rinsed three times with distilled water and allowed to dry under sterile hood for 30 min. The seeds were then plated on thirteen Petri dishes with Potato Dextrose Agar (PDA, BD, Milan, Italy) (eight seeds per plate) supplemented with 100 μg/mL chloramphenicol, 50 μg/mL streptomycin, and 50 μg/mL neomycin (Sigma-Aldrich, Milan, Italy) and incubated at 25 °C for a maximum of 10 days. The Petri dishes were examined daily, and the developed colonies were transplanted in fresh PDA plates before their margin overlapped. At the end of the incubation, contaminants such as *Alternaria* spp., *Penicillium* spp. and *Cladosporium* spp. were recognized at genus level by colony morphology and by microscopic observation [25]; the number of these fungi were counted for each plate. Key pathogens such as *M. phaseolina*, *R. solani*, *Fusarium* spp. and *Diaporthe*/*Phomopsis* complex were transferred on fresh PDA medium, analyzed under an optical microscope, and identified by microscopic structures (data not shown), while colonies of fungi with unknown morphology were transferred and further characterized. The percentage of contamination for each sample was calculated as follows: (number of seeds from which a fungal colony developed / total number of seeds in the laboratory sample) × 100.

### 2.2. Morphological Characterization of Isolates from Common Bean

Colony morphology of two isolates not resembling the typical aspect of key pathogens were named CREA OF 360.4 and CREA OF 420.36. Colonies were grown at 25 °C in the dark and the following distinctive features were recorded after ten days: spore shape and length, septa, hyphae conformation. The presence of sclerotia and microsclerotia were observed until 45 days [26] The microscopic pictures were captured with a Nikon eclipse 90i microscope (Nikon Corp., Tokyo, Japan), with 20× or 40× magnification, in transmitted light configuration. Measurements of spores and microsclerotia were done on at least fifty replicates and values were expressed as mean ± standard deviation.

### 2.3. Molecular Identification and Phylogenetic Analyses

Monosporic or monohyphal cultures of the isolates were transferred on PDA Petri dishes and grown for 7 days at 25 °C. An agar slant of 5 × 5 mm was inoculated in Potato Dextrose Broth (PDB) (BD, Becton, Dickinson and Company, Franklin Lakes, NJ, USA) and incubated at 25 °C for 72 h, with rotary shaking. The mycelia were recovered by filtration through sterile filter gauze and the biomass was collected into a mortar and ground to a fine powder in liquid nitrogen. The DNA extraction was carried out using the Genomic DNA isolation kit (Norgen, Biotek Corp., Thorold, ON, Canada), following the manufacturer’s instructions. The PCR amplification was performed using the Phusion™ High-Fidelity DNA Polymerase (2 U/μL) (Thermofisher, Foster City, CA, USA) using two following primer pairs: ITS5 (5′-GGAAGTAAAAGTCGTAACAAGG-3′) and ITS4 (5′-TCCTCCGCTTATTATGC-3′) [27]; Tef_728_ (5′-CA-TCGAGAAGTTCGAGAAGG-3′) and Tef_986_ (5-TACTT-GAAGGAACCCTTACC-3) [28]. The thermal profile was made of an initial denaturation stage at 98 °C for 1 min, followed by 30 cycles at 98 °C for 30 s, 60 °C for 30 s and 72 °C for 60 s, and a final extension at 72 °C for 10 min. Amplicons were purified by using the GeneJET PCR Purification Kit (Thermofisher, Foster City, CA, USA) following the manufacturer’s instructions. Amplicons were Sanger-sequenced in an external facility (BMR Genomics, Padua, Italy). Chromatograms were manually inspected using Chromas (http://technelysium.com.au/wp/chromas/, accessed on 1 October 2022) and used for BLASTn analyses at the NCBI database. Sequences were submitted to GenBank and can be retrieved as numbers: CREA OF 360.4 ITS: OP788183; *TEF*-1α: OP810615 and CREA OF 420.36 ITS: OP788185; *TEF*-1α: OP810616. Phylogenetic analyses were carried out for the CREA OF 360.4 and CREA OF 420.36 isolates analyzing the ITS and TEF sequences. For ITS (respectively, TEF), we employed 51 (resp. 41) publicly available sequences retrieved from the GenBank database of the National Institute of Health (NIH). IDs and relevant information are presented in the Supplementary Tables S1 and S2. Alignments were performed with MUSCLE 5 using the PPP profile alignment at default parameters [29]. Multiple alignments were trimmed using BMGE 1.12 [30] with the following settings: maximum entropy threshold: 0.5; gap rate cut-off: 0.5; minimum block size: 5. Sequences were realigned and employed for phylogenetic reconstruction using the Neighbor-Joining algorithm [31] on distances calculated with the Maximum Composite Likelihood method [32]. Bootstrap testing was based on 1000 replications. Evolutionary analyses were performed with MEGA X [33].

### 2.4. Transmissibility of the Pathogen from the Seeds to the Seedlings

In order to investigate the transmissibility of the isolates CREA OF 360.4 and CREA OF 420.36 from seeds to seedlings, seeds of healthy *P. vulgaris* landrace Dente di Morto (of the CREA gene bank) were sterilized with a solution of sodium hypochlorite (1% *v*/*v*) for 5 min, washed with sterile distilled water and dried on sterile filter paper under hood. Ten seeds per treatment were then brought in contact with the mycelium of 10-day old colony and incubated for 24 h at 26 °C and 12 h photoperiod [34,35]. The negative control consisted of seeds under the same conditions. Experiments were carried out in duplicate. Infected seeds were transferred in sand, previously sterilized twice at 120 °C for 20 min, placed in plastic boxes (11 × 11 × 3.5 cm) and incubated in a climatic chamber set at a temperature of 26 ± 2 °C, at 90% humidity and 12 h photoperiod. Watering was carried out as necessary. The pathogenicity was assessed by counting, 20 days after the sowing, the number of emerged plants and the number of diseased seedlings. For the inoculation with CREA OF 360.4, the following disease scale was adopted: 1—no symptoms; 2—crown deformity and rot; 3—basal stem deformity and/or dwarfism; 4—rotting of the seeds. Whereas, for the inoculation with CREA OF 420.36, the disease scale used was the following: 1—no symptoms; 2—30% of root rot; 3—more than 30% of root rot; 4— rotting of seeds. The disease index (*DI*), expressed in percentage, was calculated as follows:

(1) $$DI=\frac{1\times A+2\times B+3\times C+4\times D}{4\times \left(A+B+C+D\right)}\times 100$$

where *A*, *B*, *C* and *D* are the number of plants that showed the symptom levels 1, 2, 3 and 4, respectively.

### 2.5. Pathogenicity and Host Range

Pathogenicity tests were carried out by inoculating the stem of *P. vulgaris*. The seeds of the landrace Dente di Morto were sown in sterile soil and grown in a climatic chamber under a photoperiod of 12 h. After 4 weeks, the plants were inoculated with CREA OF 360.4 and CREA OF 420.36 using the V-shaped cut method, previously described by Ramirez et al. [36]. Briefly, a V-shaped cut was done on the plant stem and a plug of the mycelium was applied. The wounds were wrapped with parafilm, the plants were incubated at 26 °C for 20 days with a photoperiod of 12 h and irrigated when necessary. After 20 days of incubation, the size of the necrosis developed around the wounds was evaluated measuring the millimeters of expansion of the dry rot on the stem. The following disease scale was used for both isolates: 1—no symptoms; 2—necrosis length at the inoculation point of less than 3 mm; 3—necrotic streak of 4–6 mm around the inoculation point; 4—necrosis wider than 6 mm and/or lesion deep in the stem.

Host range experiments were conducted on several vegetable species, chosen among those of major economic importance in Southern Italy, with a focus on Fabaceae family: *Cicer arietinum* L. var. Ares (Sais Sementi, Cesena, Italy), *Pisum sativum* L. var. A Grano Rugoso Tondo (Blumen Group S.p.A., Milan, Italy), *Lens culinaris* L. local variety Lenticchia di Valle Agricola (Gene bank at CREA), *Solanum lycopersicum* L. var. Gianna (Blumen Group S.p.A., Milan, Italy), *Capsicum annuum* L. var. Quadrato d’Asti Giallo (Teraseeds s.r.l. cons, Gambettola, Italy) and *Cucumis melo* L. var. Tondo degli Ortolani. Since the stem of the legumes under testing did not allow the inoculation on the stem, the experiments were carried out by using the fungus-seed contact method as described above, whereas in the case of solanaceous and cucurbitaceous crops, the infections were carried out with the V-shaped cut method. In all cases, the DI was calculated using the Equation (1). Regarding the inoculation on tomato, the pathogenicity was evaluated as appearance of chlorotic discoloration on leaves.

Moreover, the host range was extended to *Vitis vinifera* L. to assess the pathogenicity on a common reported host for Botryosphaeriaceae. Grapevine cultivar Italia was inoculated by using detached segments of one-year old canes with the V-shaped cut method. Thirty days after inoculation, the symptoms were assessed evaluating visually the development of necrosis around the wound in the case of CREA OF 360.4, and the necrosis and evasion of *Diplodia mutila* mycelium in the case of CREA OF 420.36. The percentage of infection of the two isolates was evaluated by counting the number of branches with symptoms on the total inoculated, expressed in percentage.

## 3. Results

### 3.1. Quantification of Contaminated Seeds and Morphological Characterization of Isolated Fungi

*P. vulgaris* seeds were found to be contaminated by several saprophytic fungi such as *Alternaria* spp., *Cladosporium* spp. and *Penicillium* spp. that were reported in all the samples tested (Table 1). On the contrary, key pathogens were found in low amounts in a few samples. *M. phaseolina*, the causal agent of the soft stem rot, was found at a percentage of 1% in three varieties and the *Diaporthe*/*Phomopsis* complex was detected in 13 accessions ranging from 1 to 9% of infected seeds (Table 1). Moreover, *Fusarium* spp. were reported on eight accessions.

**Figure 1:**
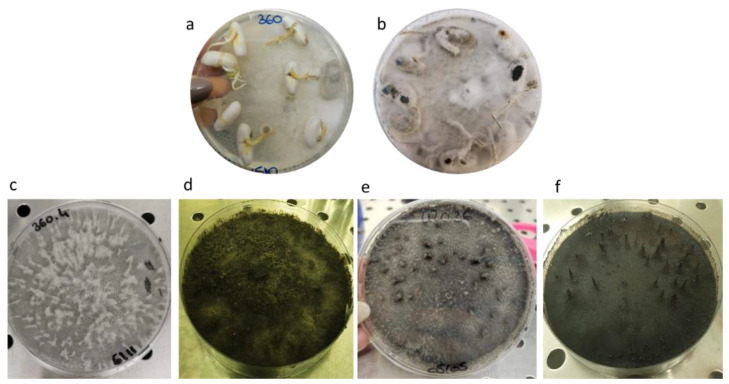
Colony morphology of CREA OF 360.4 and CREA OF 420.36. (**a**) Colony of *Botryosphaeria dothidea* CREA OF 360.4 and (**b**) *Diplodia mutila* CREA OF 420.36 developed on PDA plates after 10 days of incubation of seed samples. (**c**) Pure colony of 360.4 after 7 and (**d**) 45 days of incubation; (**e**) pure colony of 420.36 after 10 and (**f**) 45 days of incubation.

Besides common fungi (i.e., those usually isolated from *P. vulgaris* seeds), the presence of unknown colonies showing the ability to completely inhibit seed germination in PDA plates was observed. The analysis of colony development revealed a rapid growth of mycelia that also invaded and colonized initially not contaminated surrounding seeds. At the end of the incubation (10 days), all the seeds incubated on PDA plates were rotted and, where the germination took place, the rootlets appeared brown and with clear symptoms of root rot (Figure 1a,b). These colonies were transferred and cultured on fresh PDA, where they developed rapidly with abundant areal mycelium that was white in the beginning and became grey (Figure 1c,e), dark grey or black (Figure 1d,f) with age.

**Figure 2:**
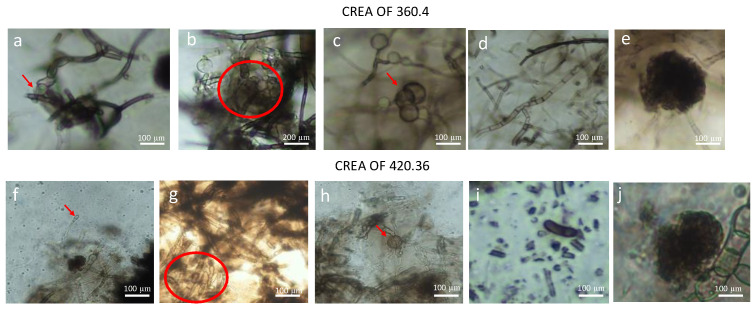
Morphological features of *Botryosphaeria dothidea* CREA OF 360.4 and *Diplodia mutila* CREA OF 420.36. In detail: (**a**–**c**,**g**,**h**) conidiogenous cells of *B. dothidea* and *D. mutila* (red circles and arrows); (**e**,**j**) sclerotia; (**f**) conidia of *D. mutila* CREA OF 420.36 on the hyphae; (**d**) right angle hyphae of *B. dothidea* CREA OF 360.4; (**i**) aseptate, dark conidia of *D. mutila*. The scale bar is reported on the bottom right corner of each panel.

The morphology was further investigated by using the optical microscope. We observed a dark and thick mycelium for both CREA OF 3640.4 and CREA OF 420.36 (Figure 2), the presence of spherical conidiogenous cells (Figure 2b,c,f–h) with an average measurement of 41.5 μm and 62.8 μm for *B. dothidea* CREA OF 360.4 and *D. mutila* CREA OF 420.36, respectively. Moreover, sclerotia were observed in both *B. dothidea* CREA OF 360.4 (average 125.39 × 99.7 μm) and *D. mutila* CREA OF 420.36 (average 123 × 105.9 μm). Finally, aseptate, dark spores were observed in *D. mutila* CREA OF 420.36 (average 26.1 × 9.1 µm), while *Botryosphaeria dothidea* CREA OF 360.4 only revealed the presence of conidia in formation (average 28.8 × 9.7 µm) attached to the hyphal tip.

### 3.2. Molecular Identification of B. dothidea CREA OF 360.4 and D. mutila CREA OF 420.36 and Phylogenetic Analysis

**Figure 3:**
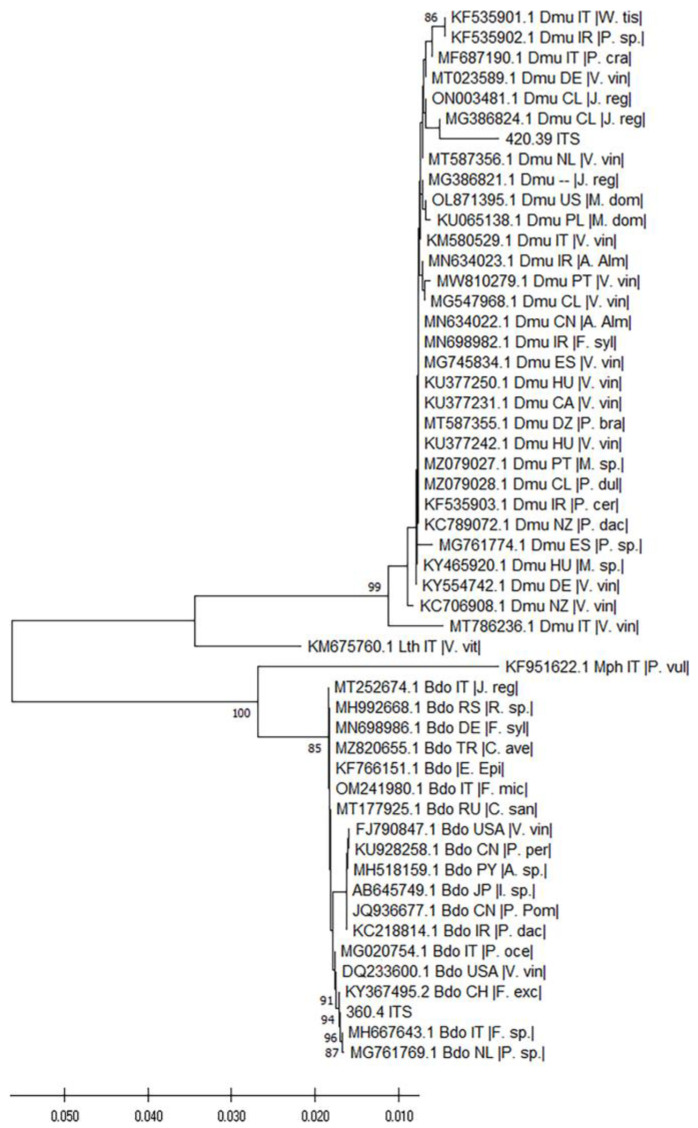
Dendrogram illustrating the evolutionary history of the isolates 360.4 and 420.36 inferred by ITS DNA sequencing. For each taxon, the graph reports the GenBank accession number, the three-letter code pathogen species, the two-letter ISO country code, and the host plant species within vertical lines (Supplementary Table S1). The percentage of replicate trees in which the associated taxa clustered together in the bootstrap test (1000 replicates) is shown if over 80. The tree is drawn to scale, with branch lengths in the same units as those of the evolutionary distances (Maximum Composite Likelihood method) used to infer the phylogenetic tree.

The molecular characterization of the two isolates was carried out by analyzing highly informative taxonomic sequences, the Internal Transcribed Spacer (ITS) and the Transcription Elongation Factor-1α (TEF). To this aim, we retrieved from GenBank homologous sequences of fungal isolates and related information (country of collection and host-species). As possible outgroup we used the sequences of *M. phaseolina* and *L. theobromae*. All the accession numbers and main information are reported in the Supplementary Tables S1 and S2. The analysis of the ITS sequences revealed that the two isolates, as expected, grouped in two well supported (>98% bootstrap values) distinct clusters (Figure 3). Specifically, isolate 420.36 clustered with the sequences annotated as belonging to *D. mutila*. Differences among these isolates were limited, except for two samples coming from the same study. The phylogenetic analysis also indicated that the isolate CREA OF 360.4 grouped among other characterized *B. dothidea* isolates. Although genetic distances were limited, the isolate under investigation is phylogenetically different from others, as indicated by the bootstrap values supporting the nodes of the branches within the large *B. dothidea* cluster. Specifically, while most of the isolates of this species grouped in two clusters, the CREA OF 360.4 and other three isolates were present in distinct branches, according to the bootstrap analysis.

**Figure 4:**
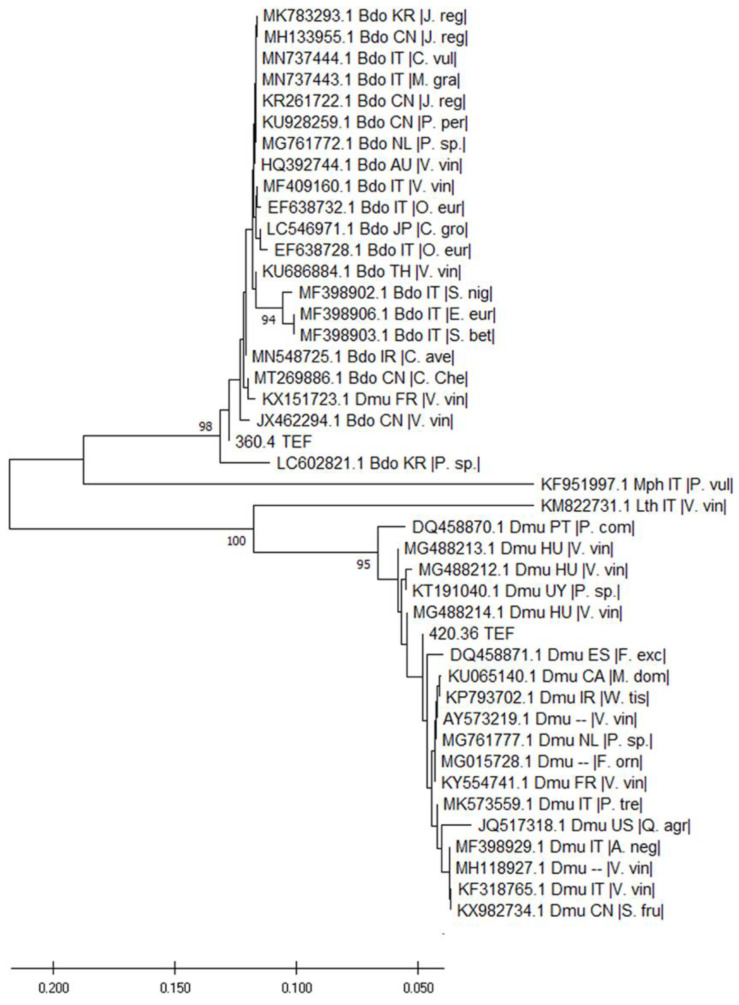
Dendrogram illustrating the evolutionary history of the isolates 360.4 and 420.36 ITS inferred by TEF DNA sequencing. For each taxon, the graph reports the GenBank accession number, the three-letter code pathogen species, the two-letter ISO country code and the host plant species within vertical lines (Supplementary Table S2). The percentage of replicate trees in which the associated taxa clustered together in the bootstrap test (1000 replicates) is shown if over 80. The tree is drawn to scale, with branch lengths in the same units as those of the evolutionary distances used to infer the phylogenetic tree (Maximum Composite Likelihood method).

The analysis of the TEF sequences also indicated that the two isolates belong to different species, as expected (Figure 4). Specifically, the two samples clustered in two well distinct groups, strongly supported by bootstrap values. The two outgroup species were also well separated. In the *D. mutila* cluster, distinctions among isolates were limited and not supported by high bootstrap values, with only two isolates coming from one study that can be considered phylogenetically distinct. The branch of the CREA OF 420.36 was supported by a low bootstrap value (33%). Within the *B. dothidea* cluster, differences were more noticeable, with two main clusters and four isolates in branches with high bootstrap support. The CREA OF 360.4 was in a single-taxon branch with a 91% bootstrap value.

### 3.3. Evaluation of the Transmissibility of B. dothidea CREA OF 360.4 and D. mutila CREA OF 420.36 in P. vulgaris from Seeds to Plantlets

**Figure 5:**
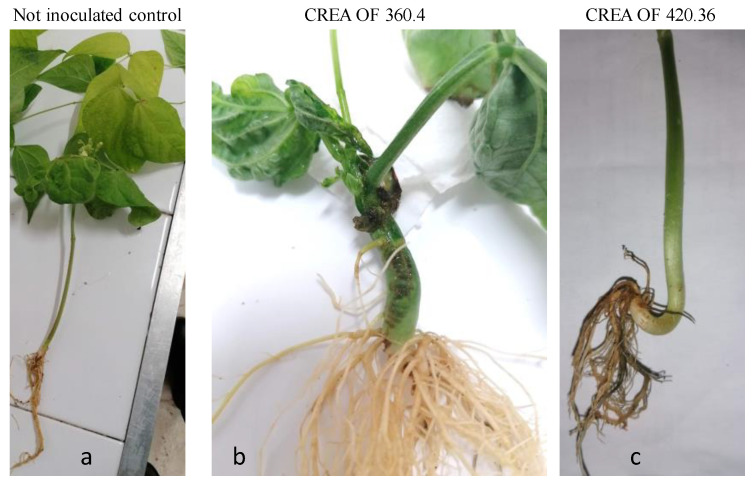
Symptoms of *Botryosphaeria dothidea* CREA OF 360.4 and *Diplodia mutila* CREA OF 420.36 on common bean upon seed inoculation: (**a**) not inoculated control; symptoms of *B. dothidea* CREA OF 360.4 (**b**) and of *D. mutila* CREA OF 420.36 (**c**) on common bean seedlings.

Seedborne pathogens are a major issue in agriculture because their ability to be transmitted to seedlings causes early death and yield loss. Thus, the transmissibility of CREA OF 360.4 and CREA OF 420.36 from the seeds to the seedlings was carefully examined. Initially, the CREA OF 360.4 isolate caused a seed rot and inhibition of germination of 50% (Figure 5). Interestingly, after five days from the inoculation, CREA OF 360.4 isolate induced reduction in plant size and the appearance of necrotic area on the cotyledons of common bean seedlings. The symptoms progressed during the days and after 20 days, the inoculated plants showed dwarfism, necrotic lesions on the stem and deformation at the crown with a disease index of 77.5% (Figure 5b). Regarding the CREA OF 420.36 isolate, it inhibited seed germination with a reduction of 50% as reported for CREA OF 360.4. During the incubation, the seedlings appeared with reduced dimensions and, after 20 days, clear symptoms of root rot were observed, for a disease index of 78.75% (Figure 5c). The results suggested the transmission of the pathogens from the seeds to the seedlings.

### 3.4. Pathogenicity Test of B. dothidea CREA OF 360.4 and D. mutila CREA OF 420.36 in P. vulgaris and Host Range

**Figure 6:**
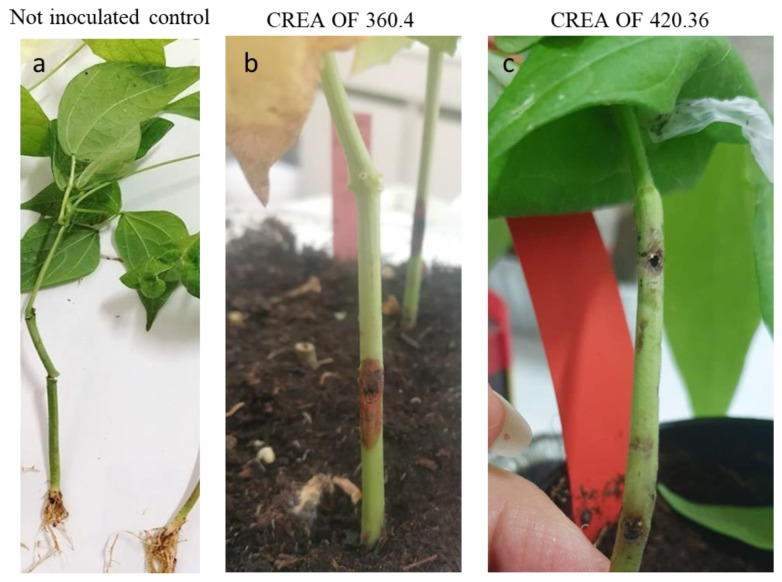
Inoculation of common bean with *Botryosphaeria dothidea* CREA OF 360.4 and *Diplodia mutila* CREA OF 430.36: (**a**) not inoculated control; (**b**) inoculation with CREA OF 360.4; (**c**) inoculation with CREA OF 420.36.

Pathogenicity tests were also conducted on three-week old seedlings of *P. vulgaris*, Dente di Morto landrace, by using a small cut on the stem to simulate a natural wound. The inoculation of CREA OF 360.4 caused the appearance of a necrotic region around the inoculation point which spread up during the experiment (DI = 57.5%) (Figure 6a). Moreover, damage to the areal part of the plants was observed with yellowing and wilting of the foliage. On the contrary, the inoculum performed with *D. mutila* CREA OF 420.36 caused the appearance of necrotic spot on the stem and the decrease in plant size (DI = 41.3%) (Figure 6b).

**Figure 7:**
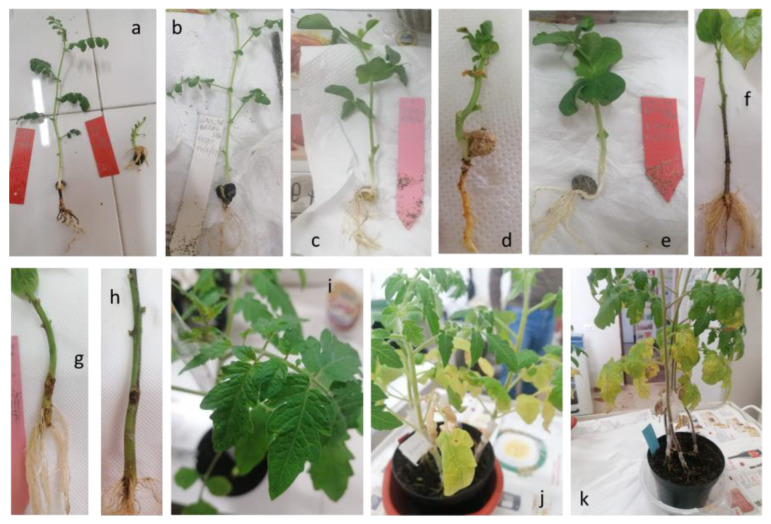
Symptoms caused by the inoculation of *Botryosphaeria dothidea* CREA OF 360.4 and *Diplodia mutila* CREA OF 420.36 on chickpea, pea, pepper and tomato plants. In detail: (**a**) Chickpea plants inoculated with *B. dothidea* CREA OF 360.4. (**b**) Chickpea plants inoculated with *D. mutila* CREA OF 420.36. (**c**) Pea non-inoculated control. (**d**) Pea plants inoculated with *B. dothidea* CREA OF 360.4 and (**e**) *D. mutila* CREA OF 420.36. (**f**) Pepper non-inoculated control. (**g**) Pepper plants inoculated with *B*. *dothidea* CREA OF 360.4 and (**h**) *D*. *mutila* CREA OF 420.36. (**i**) Tomato non-inoculated control. (**j**,**k**) Tomato plants inoculated with *B. dothidea* CREA OF 360.4 and *D. mutila* CREA OF 420.36.

To deepen our understanding of the pathogenicity of CREA OF 360.4 and CREA OF 420.36, a host range evaluation was performed including chickpea, pea, lentil, pepper, tomato and cantaloupe (Figure 7). Chickpea plants inoculated with *B. dothidea* CREA OF 360.4 showed a reduction in size and were less vigorous (Figure 7a), (DI = 76.3%) while in pea plants, dwarfing and clear symptoms of root rot were observed with a DI of 78.8% (Figure 7d). *B. dothidea* was also able to produce minor lesions on pepper stem (DI = 31.3%) and chlorosis on tomato leaves (Figure 7g,j). *D. mutila* CREA OF 420.36 caused a slight root rot in chickpea (DI = 33.8%) (Figure 7b) while in pea plants, it mildly reduced plant size, inducing a DI of 32.5% (Figure 7e) in comparison with the non-inoculated control (Figure 7c). Similarly, to the *B. dothidea* isolate, CREA OF 420.36 strain caused necrotic lesions on pepper stem (DI = 37.5%) and chlorosis in tomato plants (Figure 7h,k) with respect to the pepper and tomato non-inoculated plants (Figure 7f,i).

**Figure 8:**
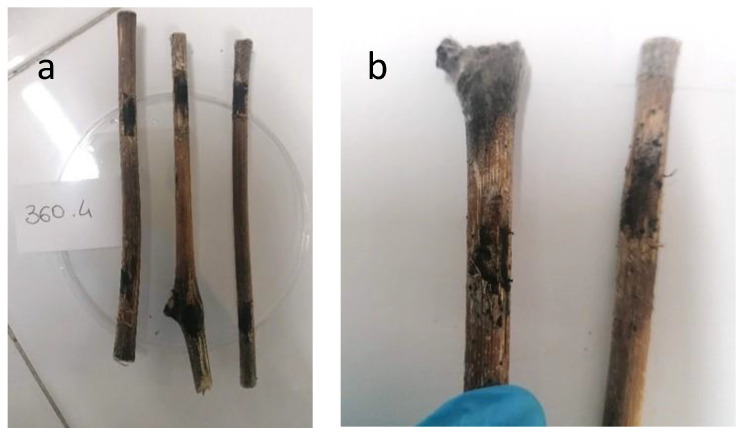
Symptoms development on one-year old canes of *Vitis vinifera* after infection with *Botryosphaeria dothidea* CREA OF 360.4 (**a**) and *Diplodia mutila* CREA OF 420.36 (**b**), isolated from common bean.

Finally, on *V. vinifera*, after the inoculation, symptoms of necrosis spread from the inoculation point throughout the canes, confirming the pathogenicity of both CREA OF 360.4 and CREA OF 420.36 (Figure 8) in the 100% and 75% of branches inoculated, respectively. Necrosis was absent in the non-inoculated control. Altogether, these findings confirmed the polyphagous nature of these pathogens even if the two isolates showed different virulence for the hosts tested.

## 4. Discussion

The increasing demand for food security and resource efficiency has led the research to focus on crops that could act as a supplement for human diet instead of animal-derived products [37,38]. In this context, legumes play a key role for their multifactorial benefits including the high content of vegetable proteins [39], the ability to solubilize nutrients in the soil [40] and their utility in agricultural rotation to decrease the impact of diseases [41,42]. The common bean is among the most cultivated legumes in the world. In Italy, there is an ample number of landraces with seeds with different shapes, colors and nutritional features [43,44,45]. Although landraces are adapted to their traditional area of cultivation environments [45], climate change and global warming may potentially lead to the insurgence of several pathological problems such as the introduction of novel pathogens in different areas. Especially for beans and other dry-seed crops, seed saving and long-term storage could spread previously overlooked, non-native or new pathogens. In this work, a screening on common bean landrace seeds has been carried out. The results highlighted the presence of commonly found contaminants such as *Alternaria* spp., *Penicillium* spp., *Cladosporium* spp. and *Fusarium* spp. In a previous work carried out on two *P. vulgaris* landraces of the Basilicata region, fungal contaminants were described, with pathogens such as *Colletotrichum lindemuthianum*, *Rhizoctonia solani* and *Uromyces appendiculatus* being the most represented [46]. Our work, however, reports the occurrence of novel pathogens included in the *Botryosphaeriaceae* family.

*B. dothidea* is a pathogen associated with grapevine decline in Iran [47], cankers on eucalypts in China [48] and with the fruit rot of apples in Italy [49]; while *D. mutila* was found as causal agent of the trunk diseases of several cultivars of grapevines in Chile [36], canker and dieback of apple trees in China [50] and the leaf blight of *Magnolia grandiflora* L. in China [51]. Here, we identified *B. dothidea* and *D. mutila* as seedborne pathogens able to compromise seed germination as well as causing seedling blight. The morphological characterization of the isolates suggested the identity of CREA OF 360.4 and CREA OF 420.36 considering the typical mycelium structures, sclerotia and conidiogenous cells commonly ascribed to the *Botryosphaeriaceae* family [52]. Nevertheless, spore formation was rarely observed in our experimental conditions. To confirm the preliminary identification, molecular analyses based on DNA sequencing in the ITS and *TEF*-*1-α* regions were performed. As expected, these two DNA sequences were suitable to give taxonomical information on *Botryosphaeria* and *Diplodia* species. The molecular characterization was deepened by studying the phylogenetic relationship, which was consistent with the colony and conidia morphology. The genetic variations among strains, evaluated calculating the genetic distance, was restricted because intra-specific sequence differences were limited, perhaps suggesting low genetic differentiation with other strains of the same species. A population-based analysis based on various co-specific isolates will be necessary to understand the possible local origin of the pathogens, also considering samples from different hosts and geographic areas.

The association of fungi of the *Botryosphaeriaceae* family with seeds has been already reported for other plant species. *B. dothidea* is a seedborne pathogen for eucalypts, where it thrives as endophyte but under favorable conditions, it causes cankers and dieback [53]. *Diplodia* spp. were described as seedborne pathogens in *Pinus* spp. for which *Diplodia pinea* was found to cause cankers and dieback of the trees [54]. Despite the wide knowledge of *B. dothidea* and *D. mutila* on woody plants, few reports have been published on their effects on legumes, but reports indicating that they can be seedborne pathogens are very scarce. The transmissibility tests showed that both *B. dothidea* CREA OF 360.4 and *D. mutila* CREA OF 420.36, besides their effects on seed germination, were also able to induce stem rot and cankers in common bean seedlings. These findings are in agreement with the results obtained by Marcenaro and Valkonen [15] who proved the presence of *Botryosphaeriaceae* in common bean seeds from Nicaragua, including *M. phaseolina* and *L. theobromae* were responsible for cankers, dieback, and seedling decline. Furthermore, the polyphagous nature of the *Botryosphaeriaceae* family has been tested through host range experiments that included legumes, solanaceous, cucurbitaceous crops and detached canes of *V. vinifera*. Interestingly, *B. dothidea* CREA OF 360.4 induced crown rot in chickpea and pea, stem canker in pepper and chlorosis in tomato plants. *D. mutila* CREA OF 420.36 caused slight crown rot in chickpea and dwarfism in pea, while slight crown rot was recorded in pepper and chlorosis in tomato. The high variability in symptoms was previously recorded in China in *Malus domestica* (Suckow) Borkh., in which *B. dothidea* is the causal agent of ring rot of the fruit and wounds on branches [55]. Similarly, *D. mutila* have been reported as the causal agent of seedling mortality in palm [56], and the dieback of *Fraxinus excelsior* L. in Sweden [57]. Altogether these findings, along with the known relationship of *Botryosphaeriaceae* with abiotic stress, make these pathogens even more threatening for a number of crops.

## 5. Conclusions

The here presented surveillance of *P. vulgaris* landraces seed health highlighted the crucial role of phytosanitary analyses for symptomless genetic resources present in gene banks. While most of the pathogen species could be expected, the detection, molecular identification and in vivo characterization of *B. dothidea* and *D. mutila* seeds indicated, for the first time, common bean seeds as host. Our results also imply that the proven ability to infect multiple host plants and the extent of the damage are important factors that expand the migration potential of these two fungal pathogens. Finally, our work also demonstrated that screening landraces is useful as it can reveal novel associations; more crucially it shows also that the uncontrolled distribution of germplasm from traditional seed collections may potentially harm the sustainability and reputation of landrace cultivation.

## Figures and Tables

**Table 1:** Percentage of seeds contaminated by fungi belonging to different genuses evaluated in 37 accessions of common bean (*Phaseolus vulgaris*).

Common Bean Accession	Percentage of Contaminated Seeds								
	*Alternaria* spp.	*Cladosporium* spp.	*Penicillium* spp.	*Fusarium* spp.	*Rhizoctonia solani*	*Macrphomina* *phaseolina*	*Collethotrichum* *lindemutianum*	*Diaporthe*/*Phomopsis complex*	Other Species
della Regina	22%	N.O. ^1^	N.O.	1%	N.O.	1%	N.O.	6%	<5%
Schiacciatello	2%	1%	N.O.	N.O.	N.O.	1%	N.O.	5%	N.O.
Tondino di Villaricca	N.O.	N.O.	N.O.	1%	N.O.	N.O.	N.O.	N.O.	N.O.
Della Regina di Gorga	N.O.	N.O.	N.O.	N.O.	N.O.	N.O.	N.O.	N.O.	N.O.
Tondino di Castel di Sasso	N.O.	N.O.	N.O.	N.O.	N.O.	1%	N.O.	N.O.	<5%
Lardari FA 38	1%	N.O.	17%	N.O.	N.O.	N.O.	N.O.	N.O.	N.O.
di Volturara Irpina FA 36	5%	N.O.	N.O.	3%	N.O.	N.O.	N.O.	N.O.	<5%
di Prata Melizzano	N.O.	N.O.	N.O.	N.O.	N.O.	N.O.	N.O.	N.O.	N.O.
Tabaccanti FA 58	2%	5%	N.O.	N.O.	N.O.	N.O.	N.O.	1%	<5%
Regina (Grottaminarda) FA 60	9%	3%	N.O.	3%	N.O.	N.O.	N.O.	9%	N.O.
della Regina (Valle dell’Angelo)	22%	N.O.	N.O.	3%	N.O.	1%	N.O.	6%	>5%
dei 7 anni	1%	N.O.	N.O.	N.O.	N.O.	N.O.	N.O.	N.O.	N.O.
Cannellino Bianco di Calitri FA 61	N.O.	N.O.	N.O.	1%	N.O.	N.O.	N.O.	4%	>5%
Tondino Bianco di Calitri FA 62	2%	N.O.	N.O.	N.O.	N.O.	N.O.	N.O.	N.O.	N.O.
Tondino di Villaricca	N.O.	N.O.	N.O.	N.O.	N.O.	N.O.	N.O.	N.O.	N.O.
Dente di Morto	11%	9%	N.O.	N.O.	N.O.	N.O.	N.O.	N.O.	<5%
Dente di Morto 2021	N.O.	N.O.	N.O.	1%	N.O.	N.O.	N.O.	N.O.	N.O.
Accession 1 Dente di Morto FA 4	26%	3%	N.O.	2%	N.O.	N.O.	N.O.	1%	<5%
Accession 2 Dente di Morto FA 5	18%	7%	N.O.	N.O.	N.O.	N.O.	N.O.	N.O.	N.O.
Accession 3 Dente di MortoFA 6	N.O.	1%	N.O.	N.O.	N.O.	N.O.	N.O.	N.O.	N.O.
Accession 4 Dente di Morto FA 4	75%	13%	N.O.	N.O.	N.O.	N.O.	N.O.	N.O.	<5%
Accession 5 Dente di Morto FA 8	71%	1%	21%	N.O.	N.O.	N.O.	N.O.	N.O.	N.O.
Accession 6 Dente di Morto FA 9	30%	27%	N.O.	N.O.	N.O.	N.O.	N.O.	1%	N.O.
Accession 7 Dente di Morto FA10	24%	2%	N.O.	N.O.	N.O.	N.O.	N.O.	1%	N.O.
Cannellino “Sessantino dei 30 anni” FA 64	N.O.	N.O.	N.O.	N.O.	N.O.	1%	N.O.	N.O.	N.O.
Fasulo a tubbettiello FA 65	N.O.	N.O.	N.O.	N.O.	N.O.	N.O.	N.O.	N.O.	N.O.
Rosso di Acerra FA 66	15%	N.O.	N.O.	N.O.	N.O.	N.O.	N.O.	2%	N.O.
Butirro	25%	8%	N.O.	N.O.	N.O.	N.O.	N.O.	N.O.	N.O.
Butirro 2021	N.O.	N.O.	N.O.	N.O.	N.O.	N.O.	N.O.	N.O.	N.O.
Bianco di Villa Santa Croce	N.O.	1%	N.O.	N.O.	N.O.	N.O.	N.O.	1%	N.O.
dei Signori	100%	N.O.	N.O.	N.O.	N.O.	N.O.	N.O.	N.O.	N.O.
della Regina (accession 2)	5%	5%	N.O.	N.O.	N.O.	N.O.	N.O.	4%	<5%
della Regina acc. 2 2021 Gandolfi	N.O.	7%	3%	N.O.	N.O.	N.O.	N.O.	N.O.	N.O.
della Regina acc. 2 2021 Mazzamauro	N.O.	N.O.	N.O.	N.O.	N.O.	N.O.	N.O.	N.O.	N.O.
della Regina acc. 2 2021 Bianco	N.O.	7%	N.O.	N.O.	N.O.	N.O.	N.O.	N.O.	N.O.
Giallo del Fortore	1%	N.O.	N.O.	N.O.	N.O.	N.O.	N.O.	1%	N.O.
Tondo Bianco	1%	15%	1%	N.O.	N.O.	N.O.	N.O.	N.O.	N.O.

^1^ N.O. = not observed.

## Data Availability

The datasets generated and/or analyzed during the current study that are not already present in the manuscript, figures, tables, and supplementary material are available from the corresponding author (L.S.) upon reasonable request.

## Supplementary Materials

The following supporting information can be downloaded at: https://www.mdpi.com/article/10.3390/pathogens12010108/s1, Table S1: Internal Transcribed Spacer 1 (ITS) sequences employed for the phylogenetic analysis. Table S2: Translation elongation factor 1-alpha (TEF) sequences employed for the phylogenetic analysis.
