# Supplementary material for: Identification and Characterization of New Seedborne Pathogens in Phaseolus vulgaris Landraces of Southern Italy
Source: Pathogens. 2023 Jan 9;12(1):108. doi: 10.3390/pathogens12010108 (PMC9866330; doi:10.3390/pathogens12010108)
Supplement: Supplementary file 1 [file pathogens-12-00108-s001.zip › pathogens-2103509-Supplementary Material.pdf]

Table S1: Internal Transcribed Spacer 1 (ITS) sequences employed for the phylogenetic analysis.

GenBank ID	Species	strain	host/isolation source	Country
AB645749.1	*Botryosphaeria dothidea*	FFPRI411072	*Illicinum anisatum*	Japan
DQ233600.1	*Botryosphaeria dothidea*	UCD1064So	*Vitis vinifera*	California
FJ790847.1	*Botryosphaeria dothidea*	UCD2467TX	*Vitis vinifera*	Texas
JQ936677.1	*Botryosphaeria dothidea*	Shi Liu Branch	*Pomegranate*	China
KC218814.1	*Botryosphaeria dothidea*	BDDP2	*Phoenix dactyliphera*	Iran
KC706908.1	*Diplodia mutila*	NZS103	*Vitis vinifera*	New Zealand
KC789072.1	*Diplodia mutila*	BSDP1	*Phoenix dactylifera*	Iran
KF535901.1	*Diplodia mutila*	KER-U-DMAPR1	*Prunus armeniaca*	Iran
KF535902.1	*Diplodia mutila*	KER-U-DMPEC1	*Prunus sp.*	Iran
KF535903.1	*Diplodia mutila*	KER-U-DMPRCE1	*Prunus cerasus*	Iran
KF766151.1	*Botryosphaeria dothidea*	CBS 115476	-	-
KF951622.1	*Macrophomina phaseolina*	CBS 205.47	*Phaseolus vulgaris*	Italy
KM580529.1	*Diplodia mutila*	1556	*Vitis vinifera*	Chile
KM675760.1	*Lasiodiplodia theobromae*	B159	*Vitis vinifera*	Italy
KU065138.1	*Diplodia mutila*	PARC153	*Malus domestica*	Canada
KU377231.1	*Diplodia mutila*	HUT69	*Vitis vinifera*	Hungary
KU377242.1	*Diplodia mutila*	HUT80	*Vitis vinifera*	Hungary
KU377250.1	*Diplodia mutila*	HUT88	*Vitis vinifera*	Hungary
KU928258.1	*Botryosphaeria dothidea*	LPPG0801	*Prunus persica*	China
KY367495.2	*Botryosphaeria dothidea*	S1_T33_L2B	*Fraxinus excelsior*	Switzerland
KY465920.1	*Diplodia mutila*	2593	*Malus domestica*	New Zealand
KY554742.1	*Diplodia mutila*	FR36	*Vitis vinifera*	France
MF687190.1	*Diplodia mutila*	ICMP:20805	*Pittosporum crassifolium*	Chile
MG020754.1	*Botryosphaeria dothidea*	IG111	*Posidonia oceanica*	Italy
MG386821.1	*Diplodia mutila*	DMnog1	*Juglans regia*	Chile
MG386824.1	*Diplodia mutila*	DMnog4	*Juglans regia*	Chile
MG547968.1	*Diplodia mutila*	EFA 467	*Vitis vinifera*	Spain
MG745834.1	*Diplodia mutila*	Bot-09	*Vitis vinifera*	Spain
MG761769.1	*Botryosphaeria dothidea*	PPO-46523	*Pyrus communis*	Netherlands
MG761774.1	*Diplodia mutila*	PPO-45196	*Pyrus communis*	Netherlands
MH500839.1	*Diplodia mutila*	UASWS1766	*Sequoiadendron giganteum*	Switzerland
MH518159.1	*Botryosphaeria dothidea*	BOTPY05	*Eucaliptus camaldulensis*	Paraguay
MH667643.1	*Botryosphaeria dothidea*	3B	*Fraxinus ornus*	Italy
MH992668.1	*Botryosphaeria dothidea*	KMI3-15	*Rubus fruticosus*	Serbia
MN634022.1	*Diplodia mutila*	IRNBS57	*Prunus amigdala*	Iran
MN634023.1	*Diplodia mutila*	IRNKB5	*Prunus amigdala*	Iran
MN698982.1	*Diplodia mutila*	NW-FVA4997	*Fagus sylvatica*	Germany
MN698986.1	*Botryosphaeria dothidea*	NW-FVA5219	*Fagus sylvatica*	Germany
MT023589.1	*Diplodia mutila*	PUCV2177	*Vitis vinifera*	Chile
MT177925.1	*Botryosphaeria dothidea*	MFLU:19-2873	*Cornus sanguinea*	Russia
MT252674.1	*Botryosphaeria dothidea*	WT7-5	*Juglans regia*	Italy
MT587355.1	*Diplodia mutila*	CBS:125.37	*Populus brabantica*	Netherlands
MT587356.1	*Diplodia mutila*	CBS:255.79	*Vitis vinifera*	Italy
MT786236.1	*Diplodia mutila*	Bt218	*Vitis vinifera*	Portugal
MW810279.1	*Diplodia mutila*	CBS 112553	*Vitis vinifera*	Portugal
MZ079027.1	*Diplodia mutila*	Bot-2018-DM196	*Malus domestica*	Chile

MZ079027.1	*Diplodia mutila*	AL8	*Prunus dulcis*	California
MZ079028.1	*Diplodia mutila*	AS7	*Prunus dulcis*	California
MZ820655.1	*Botryosphaeria dothidea*	FB1	*Corylus avellana*	Turkey
OL871395.1	*Diplodia mutila*	ICMP 13577	*Malus domestica*	New Zealand
OM241980.1	*Botryosphaeria dothidea*	FM9	*Ficus microcarpa*	Italy
OM241980.1	*Botryosphaeria dothidea*	FM9	*Ficus microcarpa*	Italy
ON003481.1	*Diplodia mutila*	F167	*Juglans regia*	Chile

Table S2: Translation elongation factor 1-alpha (TEF) sequences employed for the phylogenetic analysis.

GenBank ID	Species	strain	host/isolation source	Country
LC546971.1	*Botryosphaeria dothidea*	PF2	*Capsicum annum*	Japan
MT269886.1	*Botryosphaeria dothidea*	JZB310203	*Prunus avium*	China
MN737444.1	*Botryosphaeria dothidea*	CLRi2	*Clinopodium vulgare*	Italy
MN548725.1	*Botryosphaeria dothidea*	IRNHM-KBF22	*Corylus avellana*	Iran
MF398906.1	*Botryosphaeria dothidea*	MFLUCC_170945	*Euonymus europaeus*	Italy
KR261722.1	*Botryosphaeria dothidea*	CERC2021	*Juglans regia*	China
MH133955.1	*Botryosphaeria dothidea*	CFCC 52447	*Juglans regia*	China
MK783293.1	*Botryosphaeria dothidea*	CDH2019-2	*Juglans regia*	South Korea
MN737443.1	*Botryosphaeria dothidea*	MgPC7	*Micromeria graeca*	Italy
EF638728.1	*Botryosphaeria dothidea*	CAP233	*Olea europea*	Italy
EF638732.1	*Botryosphaeria dothidea*	CAP288	*Olea europea*	Italy
KU928259.1	*Botryosphaeria dothidea*	LPPG0801	*Prunus persica*	China
LC602821.1	*Botryosphaeria dothidea*	20-262	*Prunus armeniaca*	South Korea
MG761772.1	*Botryosphaeria dothidea*	PPO-46523	*Pyrus communis*	Netherlands
MF398903.1	*Botryosphaeria dothidea*	MFLUCC_170940	*Sambucus betulus*	Italy
MF398902.1	*Botryosphaeria dothidea*	MFLUCC_130292	*Sambucus nigra*	Italy
HQ392744.1	*Botryosphaeria dothidea*	BB158-4	*Vitis vinifera*	Australia
JX462294.1	*Botryosphaeria dothidea*	HuB10s1	*Vitis vinifera*	China
MF409160.1	*Botryosphaeria dothidea*	MFLUCC_17_0961	*Vitis vinifera*	Italy
KU686884.1	*Botryosphaeria dothidea*	MFLUCC_16_0092	*Vitis vinifera*	Thailand
MF398929.1	*Diplodia mutila*	MFLUCC_150917	*Acer negundo*	Italy
DQ458871.1	*Diplodia mutila*	JL375	*Fraxinus excelsior*	Spain
MG015728.1	*Diplodia mutila*	CAA507	*Fraxinus ornus*	unknown
KU065140.1	*Diplodia mutila*	PARC153	*Malus domestica*	Canada
MK573559.1	*Diplodia mutila*	unknown	*Populus tremula*	Italy
DQ458870.1	*Diplodia mutila*	CBS 112554	*Pyrus communis*	Portugal
MG761777.1	*Diplodia mutila*	PPO-45196	*Pyrus communis*	Netherlands
KT191040.1	*Diplodia mutila*	Fi2316	*Pyrus communis*	Uruguay
JQ517318.1	*Diplodia mutila*	UCROK1425	*Quercus agrifolia*	California
KX982734.1	*Diplodia mutila*	BJBS0001	*Malus domestica*	China
AY573219.1	*Diplodia mutila*	CBS 112553	*Vitis vinifera*	unknown
MH118927.1	*Diplodia mutila*	EFA467	*Vitis vinifera*	unknown
KX151723.1	*Diplodia mutila*	BRA08	*Vitis vinifera*	France
KY554741.1	*Diplodia mutila*	FR36	*Vitis vinifera*	France
MG488212.1	*Diplodia mutila*	T102	*Vitis vinifera*	Hungary
MG488213.1	*Diplodia mutila*	T69	*Vitis vinifera*	Hungary
MG488214.1	*Diplodia mutila*	T70	*Vitis vinifera*	Hungary
KF318765.1	*Diplodia mutila*	BL98	*Vitis vinifera*	Italy
KP793702.1	*Diplodia mutila*	KER-U-DMAPR1	*Prunus spp.*	Iran
KM822731.1	*Lasiodiplodia theobromae*	B159	*Vitis vinifera*	Italy
KF951997.1	*Macrophomina phaseolina*	CBS 205.47	*Phaseolus vulgaris*	Italy
